# The Birth Satisfaction Scale-Revised Indicator (BSS-RI)

**DOI:** 10.1186/s12884-017-1459-5

**Published:** 2017-08-29

**Authors:** Colin R. Martin, Caroline Hollins Martin, Maggie Redshaw

**Affiliations:** 1 Faculty of Society and Health, Buckinghamshire New University, Uxbridge, UK; 2 School of Nursing, Midwifery and Social Care, Edinburgh Napier University, Edinburgh, UK; 3 Policy Research Unit in Maternal Health and Care, National Perinatal Epidemiology Unit, Nuffield Department of Population Health, University of Oxford, Oxford, OX3 7LF UK

**Keywords:** Birth satisfaction, Birth satisfaction scale, Service evaluation

## Abstract

**Background:**

The current study sought to develop a short birth satisfaction indicator utilising items from the Birth Satisfaction Scale-Revised (BSS-R) for use as a brief measure of birth satisfaction and as a possible key performance indicator for perinatal service delivery evaluation.

Building on the recently developed BSS-R, the study aimed to develop a simplified version of the instrument to assess birth satisfaction easily that could work as a short evaluative measure of clinical service delivery for labour and birth that is consistent with policy documents, placing women at the centre of the birth experience.

**Methods:**

The six item Birth Satisfaction Scale-Revised Indicator (BSS-RI) was embedded within the 2014 National Maternity Survey for England. A random selection of mothers who had given birth in a two week period in England were surveyed three months after the birth. Using a two-stage design and split-half dataset, exploratory factor analysis, confirmatory factor analysis, internal consistency, convergent, divergent and known-groups discriminant validity evaluation were conducted in a secondary analysis of the survey data.

**Results:**

Using this large population based survey of recent mothers the short revised measure was found to comprise two distinct domains of birth satisfaction, ‘stress and emotional response to labour and birth’ and ‘quality of care’. The psychometric qualities of the tool were robust as were the indices of validity and reliability evaluated.

**Conclusion:**

The BSS-RI represents a short easily administered and scored measure of women’s satisfaction with care and the experience of labour and birth. The instrument is potentially useful for researchers, service evaluation and policy makers.

## Background

Placing the childbearing woman at the forefront of care provision has been paramount to the development and evolution of maternity services since the landmark *Changing Childbirth* report [1]. Reinforcing this focus on woman-centred individualised care provision has been the *National Service Framework for Children, Young People and Maternity Services* [2] and “*Maternity Matters: Choice, Access and the Continuity of Care in a Safe Service*” [3]. Direct and broad based data collection on women’s experience of care has been evident over the intervening period in the UK, with, for example, national and trust-based surveys carried out and described in *‘Towards better births’* [4] and *‘Delivered with care’* [5], followed by more recent reports [6, 7]. National studies in North America have similarly focused on women’s views of their maternity care [8, 9].

Consistent with this focus on the perinatal health and well-being of childbearing women has been recognition of the need for the development of valid and reliable measures of factors which may impact both positively and negatively on women’s experience of maternity care. Validation of a perceptions checklist for care during labour and birth and of a worries scale about labour and birth indicated that these are useful measures [10, 11]. However, satisfaction has been recognised as a key specific construct in this context [12]. The Birth Satisfaction Scale (BSS) [13] is a self-report questionnaire, targeting satisfaction with labour and birth. The original 30-item scale was developed from a thematic appraisal of the research literature, followed by the development of a 10-item short-form based on a psychometrically rigorous item-selection procedure [14]. Both forms are multi-dimensional and have been used in an international context, with validation for use in the USA [15], and translation and testing for use, for example, in a Greek population of women following childbirth [16, 17]. The BSS-R has recently been recommended as the instrument of choice for global use to assess maternal birth experience by inclusion in the *Pregnancy and Childbirth Standard Set* [18].

The views of women as consumers of maternity care are key in improving services. While the BSS and BSS-R have been shown to measure psychological dimensions of satisfaction in a reliable way in both small [14–16, 19–21] and large populations [22, 23], the potential to refine and reduce the instrument further in order to function as an *indicator* of birth satisfaction has yet to be realised. The development of such an indicator presents a number of challenges, namely reducing the number of items and simplifying the approach to scoring, while maintaining validity and reliability.

Taking the 10-item BSS-R as a starting point, using a population based survey the present study sought to develop and then determine the factor structure, validity and reliability of the shorter BSS-R indicator (BSS-RI) in order to consider this adaptation of the scale for use more widely.

The following research questions were addressed:

1. Is the BSS-RI a uni-dimensional or multidimensional measure?
2. Is there concordance of the BSS-RI with the BSS-R in terms of a tri-dimensional factor structure?
3. Do the BSS-RI and sub-scales demonstrate adequate internal consistency, divergent and convergent reliability?
4. Does the BSS-RI and sub-scales demonstrate acceptable known groups discriminant validity?

## Methods

### Design and participants

Six items from the BSS-R were selected on the basis of a review of their content and higher factor loadings (2 from each domain) [14]. These were embedded within the 2014 National survey of women’s experience of maternity care in England [7], the data set from which was used in the current study. The instrument was modified to a simplified 3-point (‘agree’, ‘agree to some degree’, ‘disagree’) scoring system with higher scores representing greater birth satisfaction (range 0–2).

Women were selected randomly by the Office for National Statistics (ONS) from birth registration records for births over a 2 week period (*N* = 10,002). Stratification of the sample was based on births in different geographical areas (Government Office Regions). Women experiencing a perinatal loss and young mothers less than 16 years of age were excluded. The ONS mailed the questionnaire directly at 3 months postpartum using a tailored reminder system [24]. The study was approved by the National Research Ethics Service committee for Yorkshire and The Humber – Humber Bridge (REC reference 14/YH/0065.

A two-stage cross-sectional design was used. Accepting that selection of a small number of items from the BSS-R may influence the conceptual alignment of the measure, and in keeping with best practice in instrument development and evaluation [25–27], a random split-half data selection procedure was adopted. The first split-half dataset (dataset one) was used to determine underlying factor structure. The second split-half dataset (dataset two) was used to confirm factor structure veracity and conduct validity and reliability evaluation of the tool.

### Statistical analysis

#### Exploratory factor analysis

Factor structure determination was accomplished using exploratory factor analysis (EFA). The multivariate and univariate characteristics of the dataset were evaluated prior to the EFA being conducted. Kline [28] advises that skew values >3 and kurtosis >10 are indicative of data non-normality. The Shapiro-Wilk’s test [29] offers a robust method to evaluate item univariate normality. Multivariate normality was evaluated using the Mardia [30, 31] and the Henze-Zirkler [32] multivariate normality tests. The principal axis factoring (PAF) factor extraction procedure was selected in view of the ordered categorical characteristics of the scoring of BSS-R items [33] as recommended for non-normal data in factor analysis [34]. The optimal number of factors was decided on the basis of parallel analysis [35] which is mathematically preferable to arbitrary determination by Eigenvalue threshold [36] and less ambiguous in interpretation compared to Cattell’s [37] scree plot. An oblimin factor rotation procedure was chosen, consistent with the likelihood that underlying factors are likely to be correlated [33, 38]. A significant item-factor loading was set at a coefficient level of 0.30 to maximise identification of candidate factor items and a coefficient level of 0.50 set to indicate a significant item-factor loading, consistent with the method of Redshaw et al. [10]. Cross-loading items were rejected to pursue simple structure.

#### Confirmatory factor analysis

The factor structure identified in EFA was evaluated in dataset two using confirmatory factor analysis (CFA) [26, 39]. Consistent with the approach taken with dataset one, the multivariate and univariate normality characteristics of dataset two were evaluated prior to the CFA [40, 41]. Model estimation procedure was predicated on the basis of data distributional characteristics [25, 26, 39]. Multiple goodness of fit tests [42] were used to evaluate the models: comparative fit index (CFI) values greater than 0.90 indicate an acceptable data fit and values of 0.95 and a good fit [43, 44]; root mean squared error of approximation (RMSEA) values of less than 0.05 indicate a good fit to the data [45]; the standardised root mean square residual (SRMR) values of less than 0.08 indicate acceptable model fit and 0.05 or less a good fit [25, 44, 46]. Model fit determination was considered almost exclusively on the basis of the indices outlined. Dataset two was similarly scrutinised regarding data distributional characteristics. Unweighted least squares (ULS) estimation would be used in the event of non-normal data and in view of the ordered-categorical scaling.

### Divergent validity

Divergent validity was determined by correlating BSS-RI scale scores with the number of weeks pregnant at the time of antenatal booking appointment. It was predicted that there would be no significant relationship between BSS-RI scores and gestation at booking.

### Convergent validity

Convergent validity was determined by correlating BSS-RI scale scores with a single overall question asking women how satisfied they were with their maternity care during labour and birth. This question was scored on a 5-point scale with anchor points ranging from very satisfied to very dissatisfied. It was predicted that there would be a significant correlation between BSS-RI scale scores and satisfaction question scores.

### Known-groups discriminant validity

Consistent with the approach taken with the BSS-R [14], known groups discriminant validity was evaluated by examining score differences as a function of delivery type (normal vaginal delivery/interventional delivery). Interventional delivery was defined by either forceps, ventouse, planned or emergency caesarean section. BSS-RI scores were predicted to be significantly higher in the normal vaginal delivery group.

### Internal consistency

An internal consistency analysis of the BSS-RI total and subscales was conducted to determine acceptability for clinical and research applications using Cronbach coefficient alpha with an alpha of 0.70 or greater being indicative of acceptable internal reliability [26, 39]. Cronbach’s alpha has been criticised for underestimating scale reliability [47] and for limitations in intrinsic measurement properties to assess reliability [48]. Consequently, McDonald’s [49, 50] omega reliability statistic was also used to estimate the general factor saturation of the test. The omega hierarchical (ω_h_) test statistic [51] has been suggested to be preferable in assessing internal reliability by providing an estimate of total scale reliability. The calculation of hierarchical and total omega values are based on specified (number of factors found by EFA) factor models derived from a minimum residual factor analysis (*MRFA*) of the dataset. The Schmid-Leiman [52] transformation procedure is thereafter performed to generate general factor loadings and from these, ω_h_ and ω_t_ are then estimated.

### Equivalence of datasets

To determine the equivalence of the two datasets, a statistical comparison of Cronbach alpha between the EFA and CFA datasets was planned using the recognised method [53]. A statistical comparison of the correlations between BSS-RI total and associated sub-scales of EFA and CFA datasets are made using an approach which assumes data distributional normality, thus Pearson’s *r* rather than Spearman’s rho correlations were used in the comparisons made [54]. The potential implications of non-normal data used with this approach are addressed in the discussion section.

Statistical analysis was conducted using the statistical software package R [55].

## Results

### Descriptive results

A survey response rate of 48% was achieved with 4578 women returning usable data. Complete 6-item BSS-R data were provided by 4201 women (<9% missing data) and used in the analyses. The average duration of pregnancy was 39.37 (SD 2.35) weeks. The majority (*N* = 4195) of women (98%) had a single baby. The majority (*N* = 3986) of women had their baby in hospital and a relatively small number (*N* = 159) at a non-hospital site midwifery-led unit or birth centre. Over half the women (59%) reported that their baby was delivered by a midwife. The mean total score of the six BSS-R items was 8.24 (SD 2.86), and the *quality of care provision*, *women’s self-assessed attributes* and *stress experienced during labour* sub-scale scores, were 3.48 (SD 0.97), 2.46 (SD 1.30) and 2.31 (SD 1.32) respectively. The random-split procedure produced dataset one for the EFA (*N* = 2096) and dataset two for the CFA and reliability and validity evaluation (*N* = 2105). The means, standard deviations, skew and kurtosis of dataset one are shown in Table 1. Examination of skew and kurtosis characteristics suggested each item to have a univariate normal distribution (skew <3, kurtosis <10), however, the Shapiro-Wilks (SW) test revealed statistically significant departure from univariate normality for all BSS-RI items (SW range = 0.53–0.81, *p* < 0.05).

**Table 1:** Individual item distributional characteristics of the Birth Satisfaction Scale-Revised Indicator (BSS-RI) from split-half data set for exploratory factor analysis (*N* = 2096)

BSS-RI item	BSS-RI Item content	Domain	Mean	SD	Skew	Kurtosis
BSS-RI 1	I was not distressed at all during labour^a^	Stress	1.00	0.77	−0.01	−1.31
BSS-RI 2	I felt very anxious during my labour and birth^b^	Attributes	1.11	0.77	−0.20	−1.31
BSS-RI 3	I felt well supported by staff during my labour and birth^a^	Quality	1.74	0.52	−1.91	2.77
BSS-RI 4	I found giving birth a distressing experience^a^	Stress	1.32	0.76	−0.62	−1.03
BSS-RI 5	I felt out of control during my birth experience^b^	Attributes	1.35	0.75	−0.66	−0.95
BSS-RI 6	The staff communicated well with me during labour^b^	Quality	1.75	0.52	−1.95	2.94

Item scoring: ^a^items ‘agree’ = 2, ‘agree to some degree’ = 1, ‘disagree’ = 0; ^b^items ‘agree’ = 0, ‘agree to some degree’ = 1, ‘disagree’ = 2

### Multivariate normality (dataset one)

The Henze-Zirkler multivariate normality test revealed a statistically significant departure from multivariate normality (HZ = 63.64, *p* < 0.05) and the Mardia multivariate normality test confirmed evidence of significant multivariate skew and kurtosis, (Mardia skew = 8.28, χ^2^ = 2894.05, *p* < 0.05, Mardia kurtosis = 60.77, Z = 29.85, *p* < 0.05).

### Exploratory factor analysis

The Kaiser-Meyer-Olkin measure of sampling adequacy (0.74) and the Bartlett test of sphericity (χ^2^ = 3918.62, df = 15, *p* < 0.001) indicated the suitability of dataset one for EFA. Parallel analysis suggested two factors, an observation confirmed by examination of very simple structure (VSS) which revealed a complexity of 0.87 with two factors. Two correlated (*r* = 0.42) factors with Eigenvalues greater than 1. (2.84 & 1.27) accounted for 55% of the variance in a common factor solution. Factor 1 loaded with the BSS-R *stress experienced* and *women’s self-assessed attributes* items and the second factor indicated by the two *quality of care* items. The item-factor loadings are shown in Table 2. The fit to data of the two-factor model was overall excellent (χ^2^_(df=4)_ = 11.35, *p* = 0.02, CFI = 0.99, TLI = 0.99, RMSEA = 0.03 (0.01–0.05, 95% CI), RMSR = 0.01). Examination of the content of items loading on Factor 1 suggested that this factor should be termed *stress experienced*.

**Table 2:** Factor loadings of the Birth Satisfaction Scale-Revised Indicator (BSS-RI) following principal axis factoring (PAF) exploratory factor analysis (split-half dataset, *N* = 2096)

BSS-RI item	BSS-RI item content	Factor 1	Factor 2
BSS-RI 1	I was not distressed at all during labour	**0.57**	0.08
BSS-RI 2	I felt very anxious during my labour and birth	**0.63**	−0.03
BSS-RI 3	I felt well supported by staff during my labour and birth	0.02	**0.85**
BSS-RI 4	I found giving birth a distressing experience	**0.79**	−0.05
BSS-RI 5	I felt out of control during my birth experience	**0.71**	0.05
BSS-RI 6	The staff communicated well with me during labour	−0.02	**0.83**

Note: Significant factor loadings in bold

### Dataset two BSS-RI item characteristics

Examination of the means, standard deviations, skew and kurtosis of dataset one (Table 3). suggested each item to have a univariate normal distribution (skew <3, kurtosis <10), however, the SW test revealed statistically significant departure from univariate normality for all BSS-RI items (SW range = 0.52–0.80, *p* < 0.05).

**Table 3:** Individual item distributional characteristics and modified domain ascription of the Birth Satisfaction Scale-Revised Indicator (BSS-RI) from split-half data set for confirmatory factor analysis (*N* = 2105)

BSS-RI item	BSS-RI item content	Domain	Mean	SD	Skew	Kurtosis
BSS-RI 1	I was not distressed at all during labour	Stress	0.97	0.79	0.05	−1.41
BSS-RI 2	I felt very anxious during my labour and birth	Stress	1.15	0.78	−0.26	−1.30
BSS-RI 3	I felt well supported by staff during my labour and birth	Quality	1.71	0.55	−1.76	2.14
BSS-RI 4	I found giving birth a distressing experience	Stress	1.31	0.75	−0.59	−1.03
BSS-RI 5	I felt out of control during my birth experience	Stress	1.31	0.76	−0.58	−1.05
BSS-RI 6	The staff communicated well with me during labour	Quality	1.75	0.52	−2.00	3.11

### Multivariate normality (dataset two)

The Henze-Zirkler multivariate normality test revealed a statistically significant departure from multivariate normality (HZ = 66.13, *p* < 0.05). The Mardia multivariate normality test confirmed evidence of significant multivariate skew and kurtosis, (Mardia skew = 7.82, χ^2^ = 2743.20, *p* < 0.05, Mardia kurtosis = 60.78, Z = 28.51, *p* < 0.05).

### Confirmatory factor analysis

**Fig. 1:**
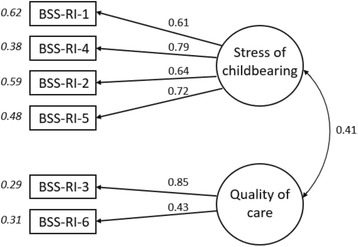
Two-factor model of the BSS-RI (For item content see Table 3.) Item-factor loadings are indicated by arrows. Variance explained within each item by the factor is indicated by the value to the left of the item box. The double-headed arrow indicates the correlation between factors. All values standardised

CFA was conducted on dataset two specifying the two-factor model identified by EFA. A single-factor version of this model was also evaluated. The two-factor model was found to be an excellent fit to data across all fit statistics. The single-factor model, by contrast, offered a poorer fit to the data. The model fit characteristics of both models are shown in Table 4. A diagrammatic representation of the two-factor best-fit measurement model with standardised item-factor loadings is shown in Fig. 1.

**Table 4:** Evaluation of the structure of the BSS-RI scale by CFA using the second split-half data set (*N* = 2105)

Model	ULSχ^2^	d.f.	CFI	TLI	RMSEA (90% CI)	SRMR
1. Two-factor	16.11	8	0.99	0.99	0.02 (0.01–0.04)	0.04
2. Single factor	70.51	9	0.95	0.92	0.06 (0.04–0.07)	0.13

*Note:* Best model fit indices from confirmatory factor analysis indicated in bold. *p* values not calculated for ULS model

*ULS* unweighted least squares, *RMSEA* root mean squared error of approximation, *SRMR* standardised root mean square residual, *CFI* comparative fit index, *TLI* Tucker–Lewis index

### Divergent validity

No significant correlation was observed between the BSS-RI total score and sub-scale scores and number of weeks pregnant at booking. The Spearman’s rho values are shown in Table 5.

**Table 5:** Spearman’s rho correlations between Birth Satisfaction Scale-Revised Indicator (BSS-RI) total scores and sub-scale scores and number of weeks pregnant at booking (*N* = 2022)

Scale	Weeks preg. *Rho*	95% CI	*p*
BSS-RI total	−0.01	−0.06 - 0.03	0.60
BSS-RI-SE	−0.01	−0.05 - 0.04	0.71
BSS-RI-QC	−0.02	−0.06 - 0.03	0.43

Key to sub-scales: *quality of care provision* (BSS-RI-QC), and *stress experienced during labour* (BSS-RI-SE)

*Note: N* = 2022 due to missing data (*N* = 83)

### Convergent validity

Correlations between BSS-RI total and sub-scale scores and the overall satisfaction with labour and birth question were all found to be positively and statistically significantly correlated and are summarised (Table 6), with significant positive Spearman’s rho correlations between BSS-RI total scale scores and BSS-RI sub-scales.

**Table 6:** Spearman’s rho correlations between Birth Satisfaction Scale-Revised Indicator (BSS-RI) total score, sub-scale scores and single item satisfaction question

Scale	BSS-RI total	BSS-RI-SE	BBS-RI-QC	Satisfaction
BSS-RI total		0.96	0.56	0.47
BSS-RI-SE			0.33	0.34
BSS-RI-QC				0.64
Satisfaction				

Key to sub-scales: *quality of care provision* (BSS-RI-QC), and *stress experienced during labour* (BSS-RI-SE). Note: Correlations with satisfaction question based on *N* = 2089 due to missing data (*N* = 16)

All correlations statistically significant at *p* < 0.01 (BSS-RI *N* = 2105)

### Known-groups discriminant validity

The mean BSS-RI total score and BSS-RI-SE and BSS-RI-QC sub-scale scores as a function of delivery type and accompanying effect sizes are shown in Table 7. The Mann-Whitney U test revealed highly statistically significant differences between groups in the direction predicted for the BSS-RI total score and the BSS-RI-SE sub-scale score. A statistically significant difference between groups was also observed in the BSS-RI-QC sub-scale score in the direction predicted. Evaluation of Cohen’s *d* revealed a small-medium effect size for the BSS-RI total score and a medium effect size for the BSS-RI-SE sub-scale. The effect size of the BSS-RI-QC sub-scale was observed to be negligible.

**Table 7:** Mean Birth Satisfaction Scale-Revised Indicator (BSS-RI) total scores and sub-scale scores as a function of delivery type (*N* = 1268, Interventional *N* = 808)

Variable	Normal	Non-normal	Mann-Whitney U	*p*	Cohen’s *d*	*d* 95% CI	Effect
BSS-RI total	8.71 (2.75)	7.42 (2.93)	645,140	<0.001	0.46	0.37–0.55	Small
BSS-RI-SE	5.21 (2.29)	4.03 (2.40)	656,180	<0.001	0.50	0.42–0.59	Medium
BSS-RI-QC	3.50 (0.95)	3.39 (1.04)	538,220	0.01	0.11	0.02–0.20	Negligible

(Standard deviations in parentheses)

### Internal consistency

Calculated Cronbach’s alpha of the BSS-RI total scale, BSS-RI-SE and BSS-RI-QC sub-scales were 0.77, 0.78 and 0.82 respectively. Evaluation of item deletion effects on Cronbach’s alpha at the BSS-RI total scale and BSS-RI-SE levels revealed no item redundancy (individual item removal reduced alpha). The BSS-RI-QC comprising just two items made item deletion evaluation inappropriate. The omega hierarchical statistic (ω_h_) was 0.61 and the omega total statistic (ω_t_) was 0.86.

### Equivalence of datasets

Comparisons between dataset one and dataset two in relation to Cronbach’s alpha for BSS-RI-Total and sub-scales are summarised in Table. 8. No statistically significant differences were revealed between the two datasets on Cronbach alpha estimations. Similarly, comparisons between datasets one and two BSS-RI-Total score and sub-scale correlations [54] are summarised (Table 9) and again reveal no evidence of statistically significant differences in the strength of associations between sub-scales and total scores.

**Table 8:** Internal consistency comparison of total and sub-scale items from EFA (*N* = 2096) and CFA (*N* = 2105) split-half datasets

Scale	EFA α (95% CI)	CFA α (95% CI)	*F*	*p*
BSS-RI total	0.77 (0.76–0.79)	0.77 (0.76–0.79)	1.00	0.92
BSS-RI-SE	0.78 (0.76–0.79)	0.78 (0.77–0.80)	1.03	0.47
BSS-RI-QC	0.82 (0.81–0.84)	0.82 (0.81–0.84)	1.00	1.00

*Note:* df1 = 2095, df2 = 2104. Confidence intervals calculation based on α to three decimals

**Table 9:** Comparison of Birth Satisfaction Scale-Revised Indicator (BSS-RI) total and sub-scale Pearson’s *r* correlation coefficients from EFA (*N* = 2096) and CFA (*N* = 2105) split-half datasets

Correlations	EFA *r* (*r*1)	CFA *r* (*r*2)	*r*1-*r*2 (95% CI)	Z	*p*
BSS-RI total – BSS-RI-SE	0.95	0.95	0.002 (−0.004–0.008)	0.64	0.52
BSS-RI total – BSS-RI-QC	0.63	0.62	0.010 (−0.027–0.047)	0.53	0.60
BSS-RI-SE – BSS-RI-QC	0.35	0.33	0.019 (−0.034–0.072)	0.70	0.49

*Note:* Confidence intervals calculation based on *r* to three decimals

## Discussion

The current study sought to develop a short, easily administered, valid and reliable birth satisfaction indicator selecting items from the BSS-R using the thematic framework supported by the original scale and the literature [12–14, 56]. Multiple concepts underpin the construct of ‘satisfaction’ with care, largely framed positively in terms of having choice and control, being informed, taking part in decision-making and having good quality, kind and respectful care, with ‘dissatisfaction’ being reflected in the negative, that is the absence of these characteristics [13, 57–59]. However, relatively few measures have been developed and appropriately tested with sufficiently large populations.

Benefitting from a large data set provided by a sample of women drawn for a national maternity survey of women’s experience of maternity care, the study used an EFA-CFA two-stage instrument development process to evaluate a two-factor model comprising sub-scales of *stress experienced during childbearing* (BSS-RI-SE) and *quality of care* (BSS-RI-QC) from which a total score (BSS-RI-Total) can also be derived and used. The psychometric characteristics of the BSS-RI and associated sub-scales appear to be excellent based on known-groups, discriminant validity, divergent validity, convergent validity and internal consistency measurement characteristics. Both datasets also appear to be equivalent in terms of key indices of internal consistency (Cronbach’s alpha) and correlations between sub-scales and total scores. Omega total (ω_t_) also revealed good total scale reliability characteristics. Thus the BSS-RI satisfies most established criteria for such a measure.

Notwithstanding the psychometric properties of the BSS-RI as described, there is a tension between the two-factor model arising from the BSS-RI, the three-factor model of the BSS-R and the three-dimensional thematic structure which underpinned the original scale. The implications in terms of the assessment of satisfaction need to be considered*.* Appraisal of the findings of the EFA and CFA and scrutiny of the 6-items which comprise the BSS-RI indicate an unambiguous two-factor structure with no evidence of cross-loading items or a ‘third factor’ that might be identified. Thus from a psychometric perspective, there is a compelling rationale for the BSS-RI to be considered as comprising two sub-scales. The data driven simplification is credible and the short six item BSS-RI represents a reframing of the conceptual model, specific and exclusive to this short-form.

With a larger pool of items and a broader response range the longer BSS and BSS-R provide a more nuanced assessment of women’s satisfaction with labour and birth care [14, 60], the BSS-R being the exemplar in terms of psychometric measurement characteristics [17, 21, 23]. The shorter BSS-RI, with high face validity, tested on a large population of women who have recently given birth, while having a distinct function as an *indicator*, clearly relates to the original conceptual content of the scale [13, 14, 56]. Our findings indicate that the BSS-RI is multidimensional, with the data demonstrating relatedness between the scales, thus providing a rationale for the use of sub-scale and total scores in measuring birth satisfaction.

### Strengths and limitations

A strength of the study was the relatively large sample size and population based sampling which facilitated the analyses conducted. This was the first study to conduct an EFA with BSS items to determine underlying factor structure. Previous insights were based on thematic content and CFA. Since the items of the BSS-RI comprised only 60% of the item pool of the BSS-R and 20% of the item pool of original scale, an EFA was essential to describe fundamental aspects of the instruments factor structure. While the brevity of the measure and ease of completion are a practical benefit in terms of response burden, the findings reveal the BSS-RI to exhibit marked deviation from univariate and multivariate normality, possibly as a consequence of simplification of the response structure. We would thus recommend the use of non-parametric statistical approaches when analysing BSS-RI data.

A potential limitation of the study concerns the number of items comprising the BSS-RI-QC sub-scale. It is suggested that a minimum number of items per factor should be three [28, 61], one rationale for this recommendation being estimation problems which may occur when sample size is small [28]. It was noted that not only was the two-factor model evaluated effectively with the large sample size used in the current study but also the appeal of a short measure such as the BSS-RI within the context of survey study designs where sample size is anticipated to be large. Additionally, sub-scales comprising two items are known to be acceptable where the items comprising the sub-scale are strongly related [14, 62] and the measure is multi-dimensional thus allowing identification with a two-item factor [61].

The response rate to the survey was modest, though similar to other surveys of women concerning their maternity care [6]. However, confidence in the findings is supported by the psychometric properties shown by the BSS-RI and the lack of variation between the two datasets, endorsing the view that women were responding to the instrument in characteristically similar fashion. A caveat in terms of the measurement aspects of the BSS-RI was that the survey design from which the data was derived did not provide an opportunity to evaluate test-retest reliability. Further evaluative work on the BSS-RI could involve assessing test-retest reliability, however, women’s views do change over time and the interval used may be critical [12, 59]. An additional strength of the current study compared to previous psychometric evaluations of the BSS and its derivatives is the availability of a convergent reliability metric against which to validate the BSS-RI. Previous studies have highlighted issues in this regard and the current study is the first to report convergent validity characteristics with respect to a satisfaction-orientated measure which remains distinct in content from the items that comprise the BSS-RI.

The measure developed, focusing on labour and birth as it does, excludes women’s antenatal and postnatal experience of maternity care. Nor does it aim to address issues of choice, information, continuity and involvement in decision-making. More broadly based analyses have the potential to facilitate the development of a measure, of which the BSS-RI could be a component, to be used across the different phases of maternity care.

## Conclusion

The current study aimed to develop a brief birth satisfaction *indicator* from the BSS-R and to establish the psychometric properties of the new measure in terms of factor structure, validity and reliability. The resulting instrument was found to have excellent psychometric qualities, with minimal burden to the women responding, while at the same time providing information that is psychologically meaningful and service delivery relevant.

This psychometrically robust indicator appears to be useful in assessing birth satisfaction, offering an evidence-based key performance indicator (KPI) for maternity services. It could be used in a range of study designs and situations, including local trust or board based surveys, serving as an effective way of monitoring women’s satisfaction with their intrapartum care, where brevity and ease of administration and scoring are key.

## Abbreviations

BSS: Birth Satisfaction Scale
BSS-R: Birth Satisfaction Scale Revised
BSS-RI: Birth Satisfaction Scale Revised Indicator
CFA: Confirmatory factor analysis
EFA: Exploratory factor analysis
HZ: Heinz Kirkler
MRFA: Minimum residual factor analysis
ONS: Office for National Statistics
RMSEA: Root mean squared error of approximation
SRMR: Standardised root mean square residual
SW: Shapiro Wilks
ULS: Unweighted least squares
